# Real-world menstrual cycle characteristics of more than 600,000 menstrual cycles

**DOI:** 10.1038/s41746-019-0152-7

**Published:** 2019-08-27

**Authors:** Jonathan R. Bull, Simon P. Rowland, Elina Berglund Scherwitzl, Raoul Scherwitzl, Kristina Gemzell Danielsson, Joyce Harper

**Affiliations:** 1 Natural Cycles Nordic AB, Stockholm, Sweden; 2 Division of Obstetrics and Gynecology, Department of Women’s and Children’s Health, Karolinska Institutet, Karolinska University Hospital, Stockholm, Sweden; 3 Department of Reproductive Health, Institute for Women’s Health, University College London, London, UK

**Keywords:** Reproductive biology, Preclinical research

## Abstract

The use of apps that record detailed menstrual cycle data presents a new opportunity to study the menstrual cycle. The aim of this study is to describe menstrual cycle characteristics observed from a large database of cycles collected through an app and investigate associations of menstrual cycle characteristics with cycle length, age and body mass index (BMI). Menstrual cycle parameters, including menstruation, basal body temperature (BBT) and luteinising hormone (LH) tests as well as age and BMI were collected anonymously from real-world users of the Natural Cycles app. We analysed 612,613 ovulatory cycles with a mean length of 29.3 days from 124,648 users. The mean follicular phase length was 16.9 days (95% CI: 10–30) and mean luteal phase length was 12.4 days (95% CI: 7–17). Mean cycle length decreased by 0.18 days (95% CI: 0.17–0.18, *R*^2^ = 0.99) and mean follicular phase length decreased by 0.19 days (95% CI: 0.19–0.20, *R*^2^ = 0.99) per year of age from 25 to 45 years. Mean variation of cycle length per woman was 0.4 days or 14% higher in women with a BMI of over 35 relative to women with a BMI of 18.5–25. This analysis details variations in menstrual cycle characteristics that are not widely known yet have significant implications for health and well-being. Clinically, women who wish to plan a pregnancy need to have intercourse on their fertile days. In order to identify the fertile period it is important to track physiological parameters such as basal body temperature and not just cycle length.

## Introduction

The menstrual cycle begins and ends with menstruation and is divided by ovulation into the follicular and luteal phases. The fertile window, during which there is a probability of conception from unprotected sex, is defined as the day of ovulation and the 5 days preceding it (the time window for sperm survival).^1^ Clinical guidelines state that a woman’s median cycle length is 28 days with most falling in the 25–30 day range and that the luteal phase is almost always 14 days long^2,3^, but there is much greater variation than this.^1,4–7^ The variation in cycle length is attributed mainly to the timing of ovulation.^4^ Nevertheless, the length of the luteal phase may also deviate significantly from 14 days. For example, the luteal phase length was between 7 and 19 days in a sample of 28 day cycles.^1^ With increasing age, cycle length reduces and the timing of ovulation becomes earlier; the variation of a woman’s cycle length reduces with age until menopause.^5,6,8,9^ Cycle characteristics also may be affected by ethnicity, high body mass index (BMI), stress and lifestyle factors.^8,10–12^ Whilst such variations in cycle parameters have previously been observed in controlled studies there is a lack of knowledge about fundamental characterisitics of the menstrual cycle in the general population.

There are more than 100 fertility awareness based (FAB) mobile apps with more than 200 million downloads^13^ and they are becoming increasingly popular for contraception^14–17^ and pregnancy planning.^18^ The FAB apps can be separated into three categories: calendar apps that look at the length of the menstrual cycle and assume average phase lengths,^19^ basal body temperature (BBT; defined as lowest resting body temperature) based apps that detect the BBT rise,^20–22^ and symptothermal apps that also measure other parameters such as cervical mucus changes.^23^ Home urinary luteinising hormone (LH) tests may also be used to determine fertile days^24^ or used as input to BBT methods to improve the accuracy of ovulation detection.^20^ All those apps relying on calendar methods to assign the fertile days assume that our historic understanding of the menstrual cycle is correct (ovulation 14 days before the next period). Available data, however, suggests that there may be significant variability in fertile days.^1,4^ Therefore, women who wish to track their fertile days for the purposes of pregnancy prevention or pregnancy planning need to understand their own cycle characteristics rather than relying on a standardised cycle.

Besides the potential benefits to the individual, fertility awareness apps and the associated databases of fertility data provide a unique opportunity to examine a large number of menstrual cycles in order to improve understanding.^25,26^ The mobile app used in the study can be used to prevent a pregnancy (‘Prevent’ mode) or plan a pregnancy (‘Plan’ mode). The mode is selected by the user during sign-up but can be changed when desired. The app uses menstruation and BBT data as inputs regardless of the mode selected by the user. The user can also add urinary LH test results, however, this is not mandatory. The automated statistical algorithm retrospectively detects the rise in BBT following ovulation and makes personalised predictions of the upcoming fertile window.^14,20,27^ With more than 1,000,000 registered users globally in August 2018, the database is one of the largest collections of menstrual cycle data ever compiled. The aim of this study is to describe menstrual cycle charactersitics observed from a large database of cycles and investigate the association of menstrual cycle characteristics with cycle length, age and BMI.

## Results

### Study population

Totally, 17.4 million non-deviating BBT measurements and 1.4 million cycles were recorded by 124,648 anonymised users of the app. They were mostly residents of Sweden, the UK and USA. Users had a mean age of 30.3 (range: 18–45) and mean BMI of 23.6 (range: 15–50). 80% started using the app with the stated intention to prevent a pregnancy and recorded an average of 8.5 cycles (5.0 ovulatory) in the study. In all, 20% started using the app to plan a pregnancy and they recorded an average of 9.0 cycles (5.6 ovulatory) in the study.

### Cycle selection

Out of 1.4 million cycles recorded by all eligible users, 3182 cycles were excluded due to pregnancy and 1886 were excluded due to being outside the 10–90 day accepted length range. Less than 1% of cycles were longer than 50 days in length. Totally, 665,603 cycles in which ovulation was not detected were excluded, of which 75% had valid temperatures entered on less than 50% of the days, which partly explains why the algorithm was unable to assign an estimated day of ovulation (EDO). An EDO was assigned in 724,134 cycles, of which 612,613 (85%) were included in the study due to having valid temperature entries entered on at least 50% of the days.

### Validation of estimated day of ovulation

The distributions of the follicular and luteal phase lengths across the study population are used to validate the app’s algorithm EDO since there is good clinical data on the expected distributions of both. We compared the distribution of follicular and luteal phase lengths in our sample of 612,613 cycles to two reference data sets: a sample of 688 cycles obtained by Baird et al.^28^ and a sample of 327 cycles obtained by Lenton et al.^7^ (Supplementary Results). The adjusted phase length distribution is a close fit to that of Baird et al. and has a slightly higher fraction of short luteal phases than that of Lenton et al.

### Cycle characteristics by cycle length

Table 1 lists the mean cycle lengths, follicular phase lengths, luteal phase lengths and bleed lengths with 95% confidence intervals (CI) in cohorts of cycles by cycle length (*n* = 612,613). Totally, 81,605 cycles (13%) were 28 days long and these cycles had mean follicular and luteal phase lengths of 15.4 and 12.6 days, respectively. Compared to the 560,078 normal length cycles (21–35 days), very short cycles had shorter bleed lengths by 0.5 days or 12% (95% CI: 0.4–0.5 days). Very long cycles had longer bleed lengths by 0.2 days or 6% (95% CI: 0.2–0.3 days). The very short cycles had shorter follicular phase by 5.4 days or 34% (95% CI: 5.3–5.5 days) and shorter luteal phases by 4.4 days or 35% (95% CI: 4.3–4.5 days). The very long cycles had longer follicular phase by 11.0 days or 66% (95% CI: 10.9–11.0 days) and longer luteal phases by 0.6 days or 5% (95% CI: 0.5–0.6 days). Less than 1% of cycles were longer than 50 days.

**Table 1:** Mean cycle lengths, bleed lengths, follicular phase lengths and luteal phase lengths in cohorts by cycle length

Cycle length range in days	Cycles (% of total)	Mean ± std cycle length in days	Mean ± std bleed length in days	Mean ± std follicular phase length in days	Mean ± std luteal phase length in days
15–20	3769 (<1%)	18.4 ± 1.6	3.5 ± 1.5	10.4 ± 2.4	8.0 ± 2.4
21–24	47,449 (8%)	23.4 ± 0.9	3.7 ± 1.4	12.4 ± 2.2	11.0 ± 2.2
25–30	395,631 (65%)	27.6 ± 1.6	3.9 ± 1.4	15.2 ± 2.5	12.4 ± 2.2
31–35	116,998 (19%)	32.4 ± 1.3	4.1 ± 1.5	19.5 ± 2.7	12.9 ± 2.3
36–50	43,240 (7%)	39.8 ± 3.7	4.2 ± 1.7	26.8 ± 4.5	12.9 ± 2.8
All cycles (10–90)	612,613	29.3 ± 5.2	4.0 ± 1.5	16.9 ± 5.3	12.4 ± 2.4

### Cycle characteristics by user age

Table 2 lists the mean cycle length, follicular phase length, luteal phase length, bleed length and per-user cycle length variation in cohorts of cycles by user age (*n* = 612,613). Cycle length decreased with increasing age with a mean difference of 2.9 days or 10% (95% CI: 2.9–3.0) between the youngest and oldest cohorts. The bleed length reduced slightly with age with a mean difference of 0.5 days or 12% (95% CI: 0.4–0.5) between the youngest and oldest cohorts. Per-user cycle length variation reduced by 0.5 days or 20% (95% CI: 0.4–0.6 days) between the youngest and oldest cohorts. The follicular phase length became shorter with age with a mean difference of 3.2 days or 20% (95% CI: 3.2–3.3 days) between the youngest and oldest cohorts. The luteal phase length varied very little between age cohorts.

**Table 2:** Mean cycle lengths, bleed lengths, per-user cycle length variations, follicular phase lengths and luteal phase lengths in cohorts of cycles by user age

Age range in years	Users (% of total)	Cycles (% of total)	Mean ± std cycle length in days	Mean ± std bleed length in days	Mean ± std per-user cycle length variation in days	Mean ± std follicular phase length in days	Mean ± std luteal phase length in days
18–24	13,391 (11%)	50,789 (8%)	30.3 ± 5.7	4.2 ± 1.4	2.9 ± 2.7	18.0 ± 5.7	12.2 ± 2.5
25–29	43,297 (35%)	209,968 (34%)	29.9 ± 5.5	4.0 ± 1.4	2.8 ± 2.7	17.6 ± 5.6	12.3 ± 2.4
30–34	41,571 (33%)	207,156 (34%)	29.2 ± 5.1	3.9 ± 1.5	2.6 ± 2.4	16.8 ± 5.2	12.4 ± 2.3
35–39	19,410 (16%)	102,553 (17%)	28.2 ± 4.4	3.8 ± 1.5	2.3 ± 2.1	15.7 ± 4.5	12.5 ± 2.4
40–45	6,948 (6%)	42,044 (7%)	27.4 ± 4.3	3.7 ± 1.5	2.4 ± 2.4	14.8 ± 4.3	12.5 ± 2.4
All cycles	124,646	612,613	29.3 ± 5.2	4.0 ± 1.5	2.6 ± 2.5	16.9 ± 5.3	12.4 ± 2.4

**Fig. 1:**
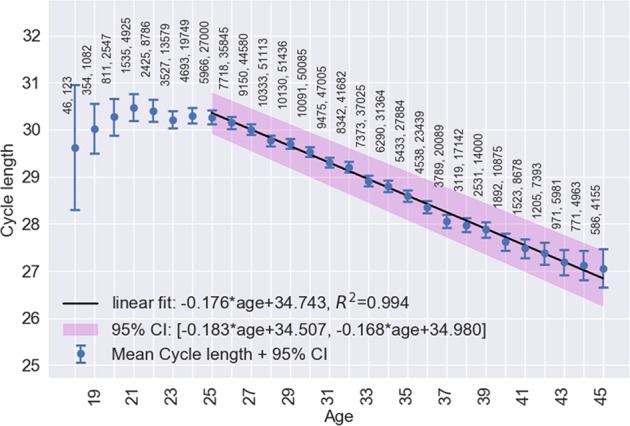
Age versus mean cycle length ±2 standard errors of the mean (blue). Linear regression (black) fitted in the range 25–45 with 95% CI (pink). Points are labelled with the number of users followed by the number of cycles

**Fig. 2:**
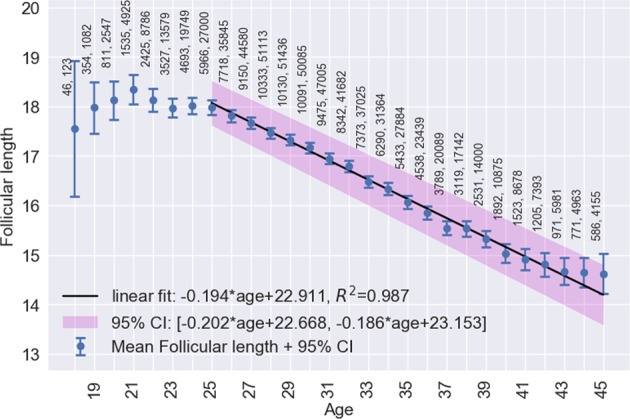
Age versus mean follicular phase length ±2 standard errors of the mean (blue). Linear regression (black) fitted in the range 25–45 with 95% CI (pink). Points are labelled with the number of users followed by the number of cycles

**Fig. 3:**
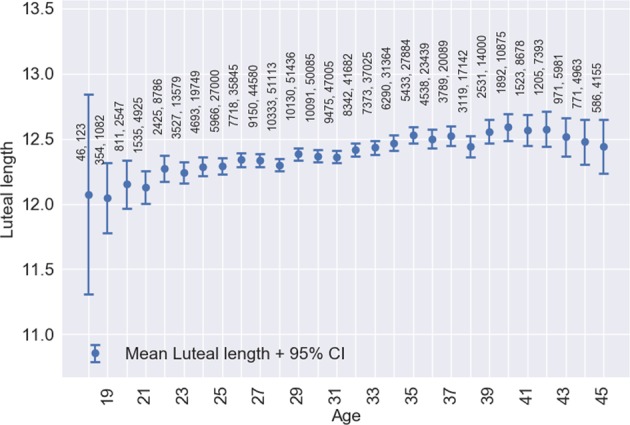
Age versus mean luteal phase length ±2 standard errors of the mean. Points are labelled with the number of users followed by the number of cycles

**Fig. 4:**
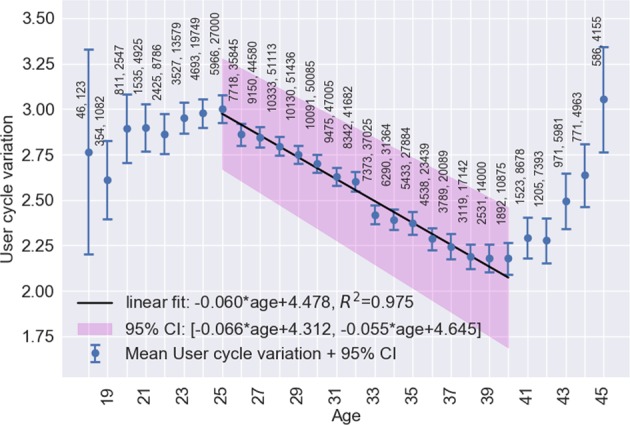
Age versus mean per-user cycle length variation ±2 standard errors of the mean (blue). Linear regression (black) fitted in the range 25–40 with 95% CI (pink). Points are labelled with the number of users followed by the number of cycles

Figures 1–4, respectively, show mean cycle length, follicular phase length, luteal phase length and per-user cycle length variation against age with error bars corresponding to 95% CI. Each point is the mean value for cycles from users of equal age and the points are labelled with the number of users. In Figs 1 and 2, linear regressions are fitted in the age range 25–45 years with 95% CI shaded in pink. Age is negatively correlated with cycle length (slope = −0.176, 95% CI: −0.168 to −0.183, *R*^2^ = 0.994) and follicular phase length (slope = −0.194, 95% CI: −0.186 to −0.202, *R*^2^ = 0.987). Mean cycle length and follicular phase length in cycles from users aged 18–24 did not fit the linear regressions. In Fig. 3, there were no significant differences between luteal phase length with age. In Fig. 4, a linear regression is fitted to the per-user cycle length variation in the age range 25–40 years with 95% CI shaded in pink. Age is negatively correlated with the per-user cycle length variation (slope = −0.060, 95% CI: −0.055 to −0.066, *R*^2^ = 0.975). Below age 25 there is little change in mean cycle length variation, but the large confidence intervals suggest that some users have a large cycle length variation. Above age 40 the variation increased markedly to its highest level of 3.1 days at age 45.

**Fig. 5:**
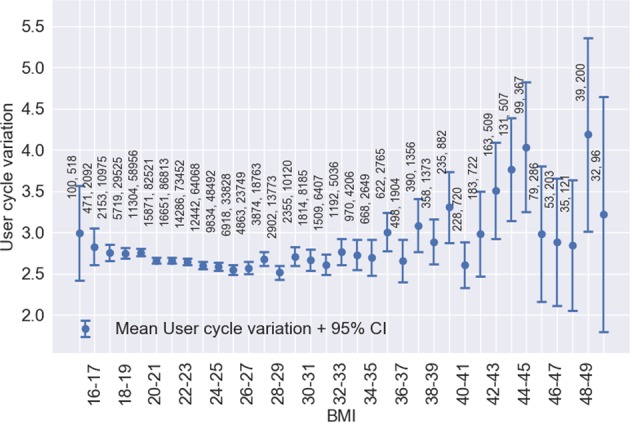
BMI versus mean per-user cycle length variation ±2 standard errors of the mean. Points are labelled with the number of users followed by the number of cycles

**Fig. 6:**
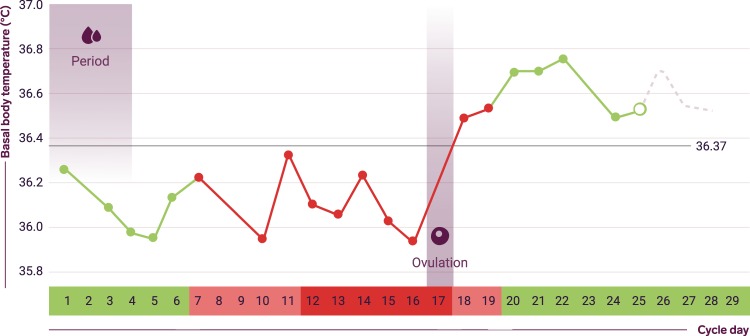
Typical temperature chart in a biphasic menstrual cycle as seen in the app. Shown here are the fertile/nonfertile days (red/green) returned by the algorithm. The fertile window days are darker red. Days with measurements are shown as filled circles. The cycle average temperature (cover line) is the grey horizontal line at 36.37 °C

### Cycle characteristics by user BMI

Table 3 lists the mean cycle length, follicular phase length, luteal phase length and bleed length in cohorts of cycles by user BMI (*n* = 612,613). The median cohort was cycles from women with normal BMI (18.5–25). Underweight women (BMI 15–18.5) had a longer mean bleed length by 0.2 days or 5% (95% CI: 0.18–0.22 days) and morbidly obese women (BMI over 35) had higher cycle length variation by 0.4 days or 14% (95% CI: 0.3–0.5 days) and longer follicular phase length by 0.9 days or 5% (95% CI: 0.8–1.0 days), than women in the ‘healthy weight’ range (BMI 18.5–25). No other clinically significant differences between BMI cohorts were observed. Figure 5 shows the mean per-user cycle length variation against BMI with error bars corresponding to 95% CI. In the BMI range 18–35 the cycle length variation is flat and above a BMI of 35 it increases although the confidence intervals are very large.

**Table 3:** Mean cycle lengths, bleed lengths, per-user cycle length variations, follicular phase lengths and luteal phase lengths in cohorts of cycles by user BMI

BMI range in kg m^−2^	Users (% of total)	Cycles (% of total)	Mean ± std cycle length in days	Mean ± std bleed length in days	Mean ± std per-user cycle length variation in days	Mean ± std follicular phase length in days	Mean ± std luteal phase length in days
15–18.5	5040 (4%)	25,735 (4%)	29.6 ± 5.2	4.2 ± 1.5	2.7 ± 2.5	16.4 ± 5.4	13.1 ± 2.1
18.5–25	83,791 (70%)	431,667 (72%)	29.3 ± 5.2	4.0 ± 1.5	2.7 ± 2.5	16.3 ± 5.3	12.8 ± 2.1
25–30	20,912 (18%)	100,228 (17%)	29.1 ± 5.2	3.9 ± 1.4	2.6 ± 2.6	16.3 ± 5.3	12.7 ± 2.2
30–35	6153 (5%)	26,483 (4%)	29.3 ± 5.6	3.9 ± 1.4	2.7 ± 2.7	16.6 ± 5.6	12.6 ± 2.1
35–50	3145 (3%)	12,011 (2%)	29.8 ± 6.0	4.0 ± 1.5	3.0 ± 3.1	17.2 ± 6.0	12.4 ± 2.1
All cycles	124,646	612,613	29.3 ± 5.2	4.0 ± 1.5	2.6 ± 2.5	16.9 ± 5.3	12.4 ± 2.4

**Fig. 7:**
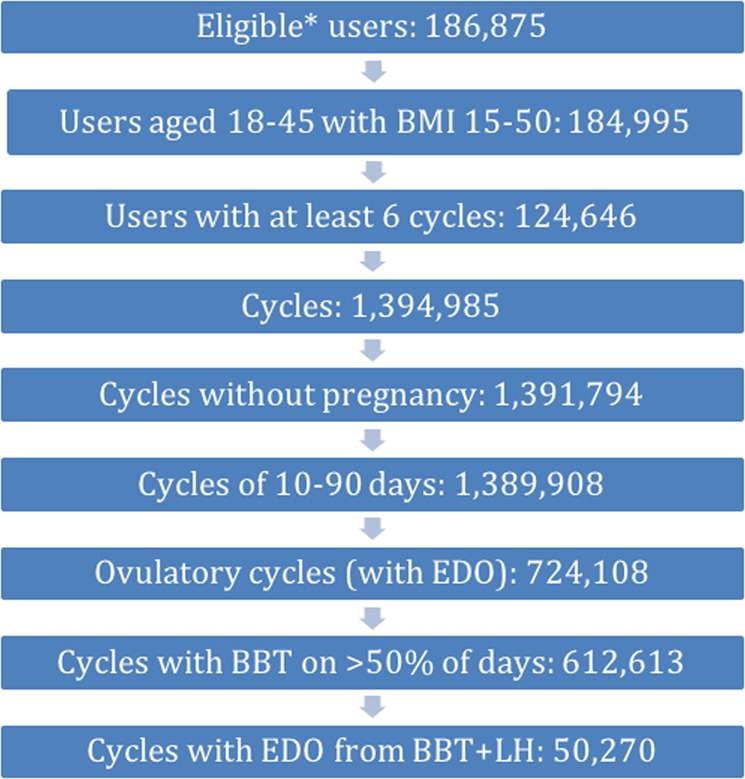
Flow diagram of user and cycle selection for study. *Eligible users met requirements on registration date, hormone use and medical conditions at the time of registration

## Discussion

In this study we analysed the key characteristics of more than 600,000 menstrual cycles. This large analysis of menstrual cycle parameters provides insight into the physiology of the menstrual cycle amongst the general population, which is not widely known. It demonstrates significant variability in cycle and follicular phase length amongst a large group of women with wide age and BMI ranges. Using this large data set, our analysis reveals important information on menstrual cycle characteristics in a real-world population of women. Knowledge and understanding of the menstrual cycle, ovulation day and the fertile period is important for both individual women and healthcare professionals providing services in reproductive health. These data are valuable for fertility educators to support educational activites around female fertility that address knowledge gaps across both the general population and the medical community.

It is a common belief that ovulation occurs on day 14 of the cycle, but our analysis has shown that for the majority of women in the real-world that this is not the case. Cycle length differences were found to be predominantly caused by follicular phase length differences (i.e., differences in ovulation day). The mean follicular phase length was 16.9 days (95% CI: 10–30). For women with a typical cycle length (25–30 days) the follicular phase length was on average 15.2 days. For women with normal but longer cycles (31–35 days), it was 19.5 days and for women with normal but shorter cycles (21–24 days) it was 12.4 days. In very short cycles (15–20 days) the mean follicular phase length was 10.4 days and in very long cycles (36–50 days) it was at 26.8 days. These findings demonstrate that the widely held belief that ovulation occurs consistently on day 14 of the cycle is not correct. Clinically, it is important that women who wish to plan a pregnancy are having intercourse on their fertile days. In order to identify the fertile period it is important to track physiological parameters such as BBT and not just cycle length.

Anecdotally most healthcare providers believe that the luteal phase is consistently 14 days in length but we found a mean of 12.4 ± 2.4 days which ranged from 8.0 days in 15–20 day cycles to 12.9 days in 36–50 day cycles. The data in this study showed that luteal phase lengths across the population do vary, albeit less than follicular phase lengths. Variation in luteal phase lengths has previously been observed in controlled clinical studies^7,24,28^; however, this is still not widely acknowledged amongst nonspecialists. The results from this study are important in order to highlight variations in phase lengths amongst the general population. It is remarkable that short cycles had a significantly reduced luteal phase relative to normal length cycles, but conversely very long cycles had a significantly long follicular phase and the luteal phase did not vary much. Out of the whole sample, 18% of cycles had luteal phases of less than 11 days. For reference, Vollman^3^ found luteal phases of less than 11 days in 15% of cycles. It has been proposed that all luteal phases of less than 10 days, and 74% of 10 day luteal phases, are abnormal^7^ implying inadequate progesterone secretion.^12^ More research is needed to ascertain whether the short luteal phases observed in this study are signs of abnormality. The use of a menstrual cycle tracking app that utilises BBT and other important physiological parameters to identify ovulation day and in turn luteal phase length can give insights into individual fertility and potentially support early identification of subfertility.

Strong linear correlations between menstrual cycle length and follicular phase length with increasing age are demonstrated. Although it is known that cycle length is likely to decrease with age, the linear correlation outlined in our analysis has never been described in such detail. The mean cycle length dropped by 3.2 days from age 25 to 45 and the mean follicular phase length dropped by 3.4 days in the same period. Above 40 the variation increased dramatically. These results are in alignment with those of reference studies.^3,6,9^ The mean bleed length of 4.0 ± 1.5 days, and its decline by 0.5 days from 4.2 ± 1.4 days in women aged 18–24 to 3.7 ± 1.5 days in women aged 40–45, is in agreement with Harlow.^12^

It is well-established that obesity is related to menstrual disorders, infertility, miscarriage, obstetric complications, live birth rate and can affect the success of assisted reproductive technology.^29–32^ In our study, we were not able to demonstate significant effects of BMI on ovulatory menstrual cycle characteristics. This is likely due to underrepresentation of women with high BMI within the study population. The strongest effect seen was an increase of per-woman cycle length variation of 14% in women with BMI of 35–50 relative to women with normal BMI. This effect is expected because pre-existing medical condition (PCOS) is associated with obesity and causes erratic menstrual cycles.^10^ Future research may investigate the effect of BMI on cycle characteristics in greater depth.

The main limitation of this study is that the study population is derived solely from users of the app who may not be representative of the wider population. In particular, only 8% of women in our study were obese compared to 15% of women in the general population.^33,34^ Only cycles with ovulation detected were included in the study. Of the 1.4 million cycles initially considered, ovulation was not detected in 665,603 (48%) of which most did not have sufficient BBT measurements to enable detection. Nevertheless, there is a bias caused by excluding these cycles. The incidence of ovulatory cycles recorded was lower among users with BMI of 30–35 (49% ovulatory) and 35–50 (45% ovulatory) compared to users with normal BMI (53% ovulatory). We also acknowledge the potential for human error in identification of the start of the cycle, the start and peak of the LH surge and the BBT rise based on self-reported bleeding, urinary LH test results and temperature measurements respectively. Study participants were able to purchase approved LH tests from the app developers, however, it is known that some users prefer to buy other commercially available tests between which there may be small variations in LH threshold values for a positive result. Measuring BBT and LH every 24 h limits the precision of phase length calculations.

Given the variations in cycle length and follicular phase length that we have described, especially for cycles outside the average range (25–30 days), an individualised approach to identification of the fertile window should be adopted. There are more than 100 fertility tracking apps freely available for download. Many of these apps claim to identify fertile days based on traditional assumptions about key menstrual cycle parameters such as regularity of cycle length, follicular phase length and luteal phase length. Apps giving predictions of fertile days based solely on an outdated understanding of ovulation day variation could completely miss the fertile window. It is, therefore, unsurprising that several studies have shown that calendar apps are not accurate in identifying the fertile window.^35–38^ This study has demonstrated that such assumptions are invalid and that in reality there are significant variations in several key parameters in the general population.

Some fertility apps are based on sophisticated algorithms for individualised identification of the fertile window relying on physiological parameters such as BBT which are more acceptable for large numbers of women.^14,27^ Whilst LH test kits have been used to determine the day of ovulation for decades, they have a significant margin of error when used in isolation. The addition of BBT and the use of a fertility app may help to narrow down testing days and therefore be more convenient and cheaper. Individualised identification of the fertile window based on BBT and menstruation dates can help to reduce the time to conception in some cases.^18^

With women globally delaying fertility^39^ the potential value of fertility tracking apps as a platform for delivery of individualised fertility education and preconception care should not be underestimated. Anecdotally there is poor understanding of fertility amongst the general population, which can lead to both unintended pregnancies and delayed time to conception with associated psychological suffering for those wishing to start a family.^40^ Fertility education delivered through an app has the potential to improve doctor–patient interactions^41^ and communication between partners. The value of fertility apps as educational platforms to achieve public health benefits through standardised health promotion messages during key stages of reproductive life such as preconception, pregnancy and birth spacing is also being explored.

Finally, the widespread use of mobile phone apps for personal health monitoring is generating large amounts of data on the menstrual cycle. Provided that the real-world data can be validated against traditional clinical studies done in controlled settings, there is enormous potential to uncover new scientific discoveries. This is one of the largest ever analyses of menstrual cycle characteristics. These initial results only scratch the surface of what can be achieved. We hope to stimulate greater interest in this field of research for the benefit of public health.

## Methods

### Menstrual cycle data collection

Physiological data, including daily BBT (sublingual measurement), cycle by cycle dates of menstruation, and urinary LH test results, were collected prospectively from users of the Natural Cycles app. Participant characteristics including age and BMI were determined through mandatory in-app questions that must be completed during the sign-up process. Users are recommended to measure their temperature on 5 out of 7 days per week as soon as they wake up. They are requested to report whether a temperature measurement may be deviating for reasons such as disrupted sleep or alcohol consumption the night before. The algorithm also identifies deviating temperatures if the value is outside the range 35.0–37.5 °C.

All users in the study had consented at registration to the use of their data for the purposes of scientific research and could remove their consent at any time. This study was a subanalysis of data collected as part of a wider study protocol that was reviewed and approved by the regional ethics committee (EPN, Stockholm, diary number 2017/563-31).

### Identification of ovulation day

A surge in LH is responsible for triggering follicle rupture.^2^ The start of the surge is approximately 28–48 h before follicle rupture and peak LH levels are reached 12 h before follicle rupture.^42^ After follicle rupture the corpus luteum forms, marking the start of the luteal phase, and secretes progesterone for the duration of the luteal phase in order to prime the endometrium for embryo implantation.^2^ Elevated levels of LH are detectable in blood and urine samples. At the onset of menses, marking the start of the follicular phase, the corpus luteum collapses and progesterone levels fall back to a low level until the next preovulatory increase. Progesterone has a thermogenic effect so its levels can be tracked by measuring BBT. BBT is at a relatively constant low level during the follicular phase, reaching its lowest level (the nadir) prior to ovulation,^43^ and then displays a distinct rise of 0.2–0.3 °C following ovulation.^44^ The higher level of BBT is sustained during the luteal phase before falling back to the lower level at the start of the next cycle.^44,45^

The algorithm within the app detects ovulation retrospectively based on BBT measurements, menstrual cycle parameters and additionally on positive urinary LH tests. The algorithm can identify the BBT rise associated with ovulation in the presence of measurement errors, missing data and BBT rise occurring over a variable length of time.^20^ The risk of misidentification is reduced by excluding deviating temperatures. In order to determine that ovulation has occurred, as a minimum requirement the rolling average BBT (average of valid (nondeviating) temperatures over the last three calendar days) must be higher than both the woman’s follicular phase average and her cover line (the average temperature across all data entries) and consistent with her luteal phase average. Figure 6 illustrates a typical biphasic temperature graph in an ongoing ovulatory cycle from a user in ‘Prevent’ mode. The horizontal grey line is the cover line. Comparisons are made using standard statistical techniques taking into account sample size and standard deviation. If ovulation is not detected in this initial test then more tests are performed with a rolling average over an increasing number of days up to 1 week. Ovulation detection is less likely if there are valid temperature measurements on fewer than about 50% of cycle days.

If a positive-LH test has been recorded, fewer high temperatures are required in order to detect ovulation since the LH test provides extra confidence that ovulation has occurred. The app recommends which days to take an LH test, considering the uncertainty of the ovulation day such that it minimises the number of LH tests used while ensuring that the user will not miss her surge. If the user is in Prevent mode, the algorithm only recommends to check for LH if the user had at least three cycles off hormonal contraception and the total ovulation uncertainty is less than ±10 days. For users on Plan mode the app always recommends which days to check for LH since Plan users are in general more keen on finding the surge, even if it requires a large number of LH tests. The app will, however, only recommend to start checking LH 10 days prior to the earliest recorded ovulation day even if the total uncertainty is larger.

As the LH surge typically lasts for several days^42^ the probability of missing the surge if only testing every other day is relatively small. The app, therefore, recommends to only test every other day until close to the expected ovulation day. If one positive LH test has been entered, but no positive or negative LH test entry exists on the day immediately before, then the user is encouraged to test the following day to establish whether the positive test corresponds to the first or second day of the surge. If no such test is entered, the app assumes the first LH test marks the first day of the surge.

Cycles in which ovulation has been detected are hereafter referred to as ovulatory cycles. If ovulation has been detected in the current cycle then the algorithm selects the most suitable candidate day to call the *First High Point* (FHP) using a system of measurements based on comparisons of each temperature to the phase averages. This is the day on which the temperatures immediately before and after are most consistent with the follicular and luteal phase averages respectively. On average the FHP temperature is just below the cover line. In a previous study the FHP was 1.9 ± 1.4 days after the estimated start of the LH surge,^20^ similar to a comprehensive study of different markers of ovulation by Ecochard et al.^45^ where the FHP was most often 2 days after the LH peak. An evaluation of the timing of the FHP and the LH peak relative to the data of Ecochard et al (2001) is available in Supplementary materials.

In a clinical study^43^ the FHP was observed in most cycles during the 12 h following ovulation. Because users of the app only measure temperature every 24 h, the FHP is expected to be detectable by the algorithm the day after ovulation. This means that ovulation itself is estimated to occur on the day of the last low temperature before the rise as suggested by Hilgers and Bailey^46^ and Mouzon et al.^43^ We define the EDO as the day before the FHP, the day of the last low temperature. According to convention the follicular phase ends on the EDO and the luteal phase starts the day after the EDO.

Another marker besides the BBT shift that has been used in clinical settings to estimate the day of ovulation is the day of luteal transition (DLT) defined as the ratio of oestrogen to progesterone falling below a critical threshold.^28,47^ The DLT ovulation detection algorithm has been designed to coincide with the peak of the LH surge.^47^ Although DLT is not intended for home use we mention it here because a study using it will be used as a source of reference data for validating the results of this study.

### Inclusion/exclusion criteria

Women using the app who had registered between 1st September 2016 and 1st February 2019, had given their consent for the use of their data in research, were aged 18–45 at registration, had a BMI between 15 and 50 and had not been using hormonal contraception within the 12 months prior to registration were included. Users who stated at registration that they had a PCOS (hypothyroidism or endometriosis) or who had menopausal symptoms were excluded. They were required to have logged at least ten nondeviating temperatures.

Cycles were, included if they were recorded by a user with at least six complete cycles (with or without detected ovulation), the cycle length was between 10 and 90 days and nondeviating temperatures had been recorded on at least 50% of cycle days. ‘Non-deviating temperatures’ are defined as temperature measurements where the user has not selected the temperature to be abnormal (e.g., due to unexplained fever or high alcohol intake) when entering into the app. Cycles were excluded if a pregnancy was reported by the user or was otherwise flagged as possibly pregnant by the algorithm due to a significantly longer luteal phase than the user’s average and sustained high temperatures. Figure 7 summarises the number of users and cycles at each step of the selection process.

### Study design

The ‘normal’ menstrual cycle is conventionally classified as 21–35 days in length, frequent menstrual bleeding (polymenorrheic) cycles as being under 21 (very short cycles) and infrequent menstrual bleeding (oligomenorrheic cycles) as being over 35 days (very long cycles).^48^ In this study bleed length was defined as the number of consecutive days on which bleeding—not spotting—was recorded. Spotting is defined as very light bleeding (a few drops of blood) or brown/pink fluids. Users are instructed not to log very light bleeding just before the period as bleeding but to wait until the flow increases. The follicular phase was defined as the first day of recorded menstruation to the EDO. Luteal phase length was defined as the day after the EDO to the day before the next day of recorded menstruation. The per-user cycle length variation was defined as one standard deviation of a user’s cycle lengths.

We calculated mean cycle length, duration of bleeding (bleed length), follicular phase length and luteal phase length in ovulatory cycles. The following cohort splits by cycle length were defined: very short cycles (15–20 days), short cycles (21–24 days), medium cycles (25–30 days), long cycles (31–35 days) and very long cycles (36–50 days). We calculated the same statistics as well as per-user cycle length variation for cohorts of ovulatory cycles by user age at registration (18–24, 25–29, 30–34, 35–39 and 40–45 years) and BMI (15–18.5, 18.5–25, 25–30, 30–35 and 35–50). We also calculated the mean proportion of ovulatory cycles as a fraction of all cycles recorded by the user in each of the age and BMI cohorts.

Owing to the very large sample sizes in this study, *P* values were not calculated since they can be very small even if differences between cohorts are of no clinical significance.^49^ Instead, effect size between two cohorts was estimated as a mean difference with a 95% confidence interval calculated from 200 bootstrapped cohort-sized randomly selected samples with replacement.^50^ Mean differences are also given as a percentage of the mean in the combined cohorts. Where linear regression is used, we quote the coefficient of the slope with a 95% confidence interval and *R*^2^ value.

### Reporting summary

Further information on research design is available in the Nature Research Reporting Summary linked to this article.

## Supplementary information

Analysis of Luteal Phase Distributions Study Population vs Published Reference Material

Reporting Summary

## Data Availability

The data that support the findings of this study are available from Natural Cycles Nordic AB but restrictions apply to the availability of these data, and so are not publicly available. Data are, however, available from the authors upon reasonable request and with permission of the developers.

## Supplementary information

**Supplementary Information** accompanies the paper on the *npj Digital Medicine* website (10.1038/s41746-019-0152-7).

## Code availability

The code that constitutes the mobile application (including the ovulation detection algorithm) is commercially sensitive and not available for release. The code used to analyse the database of recorded cycles may be made available upon reasonable request to the corresponding author and with permission of the company.
