# Supplementary material for: Real-world menstrual cycle characteristics of more than 600,000 menstrual cycles
Source: NPJ Digit Med. 2019 Aug 27;2:83. doi: 10.1038/s41746-019-0152-7 (PMC6710244; doi:10.1038/s41746-019-0152-7)
Supplement: Supplementary file 1 — Analysis of Luteal Phase Distributions Study Population vs Published Reference Material [file 41746_2019_152_MOESM1_ESM.pdf]

**Supplementary Results**

**Analysis of Luteal Phase Distributions Study Population vs Published Reference Material.**

The distributions of the follicular and luteal phase lengths across the study population are used to validate the Natural Cycles algorithm's EDO since there is good clinical data on the expected distributions of both. We compare the distribution of follicular and luteal phase lengths in our sample of 612,613 cycles to two reference data sets: a sample of 688 cycles obtained by Baird et al[28] and a sample of 327 cycles obtained by Lenton et al.[7] Baird et al estimated the day of ovulation using an algorithm for computing the DLT which is expected to coincide with the peak of the LH surge.[47] Lenton et al used the peak of the LH surge, measured from daily blood and urine samples, to estimate ovulation day.

In order to compare phase lengths, we correct for the difference in definition of EDO between the current and reference data. We analyse a subset of 50,270 cycles from the current data set with 'highest quality' LH data, namely in which a negative LH test was followed the day after by a positive LH test or by 2 positive LH tests on the 2 following days. Cycles with 3 or more consecutive positive tests or with positive tests separated by a day or more are not included in the subset. These patterns of LH tests give confidence that the start of the surge has not been missed. It is assumed that the first positive LH test marks the peak of the surge. The interval between the first positive LH test and the FHP from the algorithm in the subset of 50,270 cycles was 1 day in 39% of cycles, 2 days in 32% of cycles, and between 0 and 3 days in 86% of cycles, similar to the results of a previous study (where the FHP was called ovulation) and a somewhat narrower range than was found between the LH peak and the FHP by Ecochard et al.[45] A figure comparing the distribution of the interval to

the data of Ecochard et al is in Supplementary Materials. We define the *Corrected Day of Ovulation* (CDO) - an estimate of the LH peak in the whole sample of 612,613 cycles - as the EDO minus the estimated LH test-EDO interval. This correction is made to align the definition of ovulation day with that used in reference studies[7, 47] in order to make a comparison of luteal phase length distributions. The LH test-EDO interval is estimated in cycles without LH data by random selection with replacement from the interval distribution in the subset of 50,270 cycles. That is, we assume that the interval distribution in the whole sample is identical to the distribution in the subset of cycles with LH data. The adjusted luteal phase length, defined as starting the day after the CDO, is directly comparable to Baird et al and Lenton et al.

Table 1 lists the mean, mode, range and percent of phase lengths within ±4 days of the mode for both the unadjusted and adjusted luteal phase lengths in the whole sample (n=612,613) and the same statistics from Baird et al. The adjusted phase length statistics are in good agreement with the reference data: the modal value is 13 but 14 day phases are almost as common. The adjusted mean length is 13.3 days, 0.9 days longer than the unadjusted mean length. Figure S1 shows the distribution of adjusted luteal phase length (n=612,613) and the reference distribution from Baird et al. The adjusted phase length distribution is a close fit to that of Baird et al and has a slightly higher fraction of short luteal phases than that of Lenton et al.

These results provide confidence that on average, the first positive LH test represents the LH peak well. Furthermore, the FHP (hence EDO) has a well defined relationship to the LH peak and the two markers are more tightly coupled than the analysis of Ecochard et al shows.

(Figure S2) In the rest of the paper we report unadjusted phase lengths - the follicular phase ending on the EDO and the luteal phase starting after the EDO - which are expected to be well correlated with the results of studies using clinical techniques for detecting ovulation.

| Data Set | Baird et al (1995) | Natural Cycles | |
|---|---|---|---|
| Luteal Phase Definition | Starts after EDO from DLT algorithm | Starts after EDO from Natural Cycles algorithm | Starts day after CDO* |
| Cycles | 498 | 612,613 | 612,613 |
| Mean ± std. dev. | 13.1 ± 2.2 | 12.4 ± 2.3 | 13.3 ± 2.2 |
| Mode | 14 | 13 | 14 |
| % ± 4 days of mode | 77 | 93 | 96 |
| Range | 5-20 | 3-30 | 3-30 |

Table 1. Adjusted and unadjusted luteal phase characteristics from 612,613 cycles from the Natural Cycles database compared to a clinical study of 688 cycles (Baird 1995). Unadjusted luteal phase starts the day after the EDO and adjusted luteal phase starts the day after the CDO. *CDO: corrected day of ovulation, estimate of LH peak.

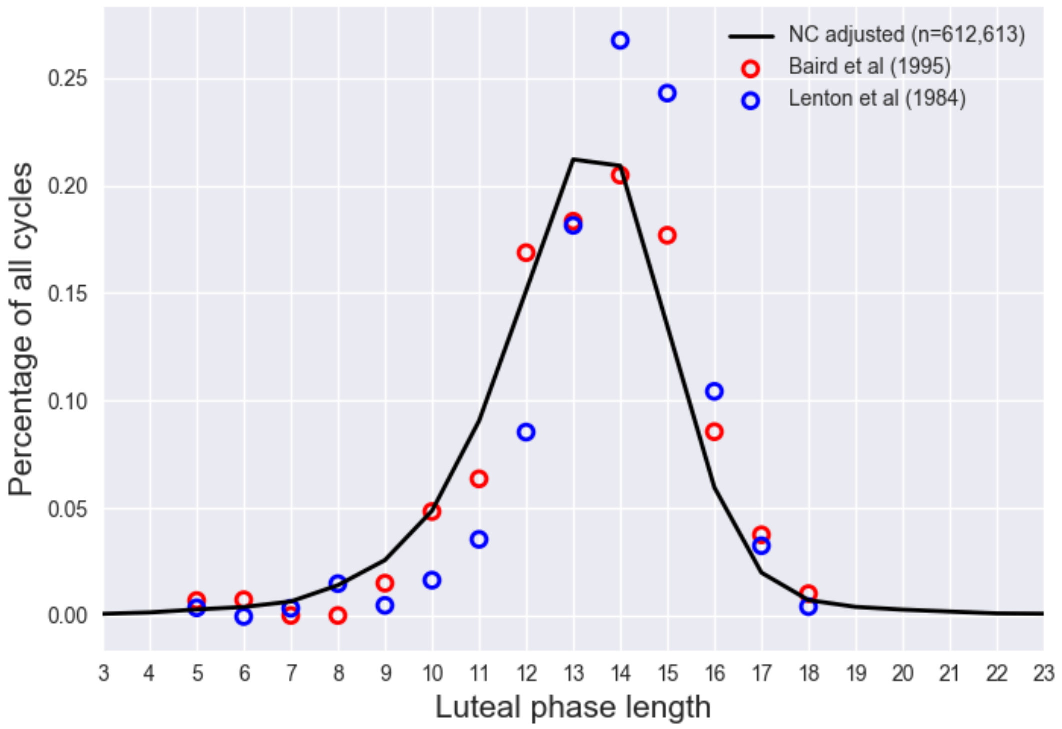


Figure S1. Luteal phase length distributions. Red circles: Baird et al (1995); blue circles: Lenton et al (1984); black: Natural Cycles adjusted luteal phase length (n=612,613). The adjusted luteal phase length starts the day after the CDO which is an estimate of the peak of the LH surge.

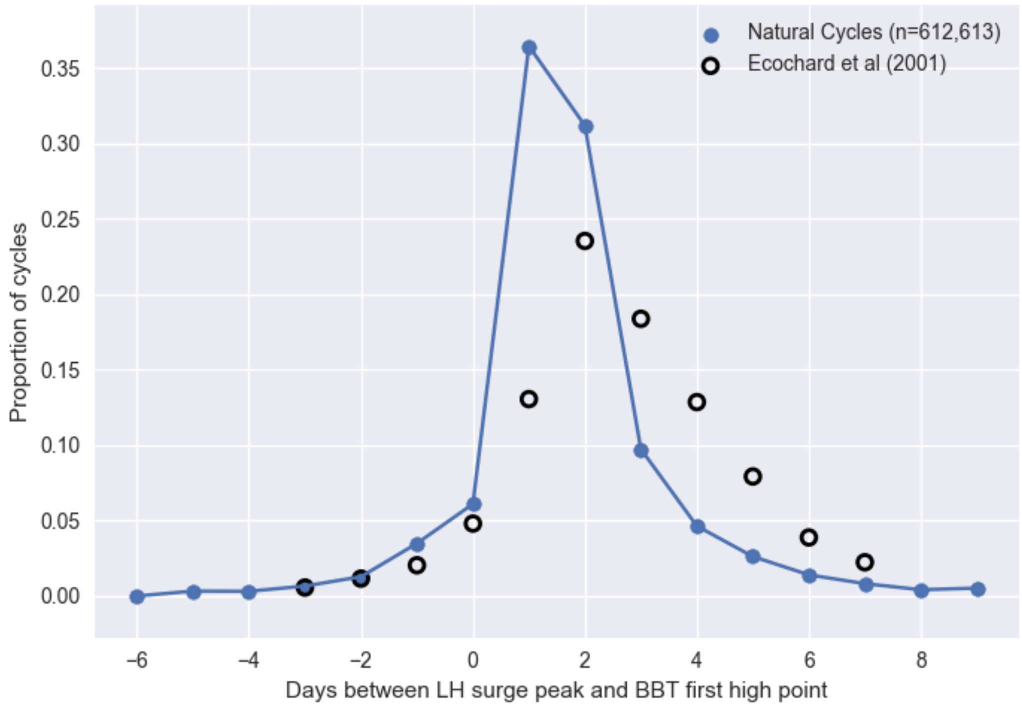


Figure S2. Interval from LH surge peak to BBT first high point in the Natural Cycles data (n=612,613) and compared to Figure 4 in Ecochard et al (2001). In the Natural Cycles data the first positive LH test denotes the LH surge peak in a sample of 50,270 cycles with LH data.
